# RAIDD mutations underlie the pathogenesis of thin lissencephaly (TLIS)

**DOI:** 10.1371/journal.pone.0205042

**Published:** 2018-10-03

**Authors:** Hyun Ji Ha, Hyun Ho Park

**Affiliations:** College of Pharmacy, Chung-Ang University, Seoul, South Korea; The University of Texas MD Anderson Cancer Center, UNITED STATES

## Abstract

Abnormal regulation of caspase-2-mediated neuronal cell death causes neurodegenerative diseases and defective brain development. PIDDosome is caspase-2 activating complex composed of PIDD, RAIDD, and caspase-2. Recent whole-exome sequencing study showed that the RAIDD mutations in the death domain (DD), including G128R, F164C, R170C, and R170H mutations, cause thin lissencephaly (TLIS) by reducing caspase-2-mediated neuronal apoptosis. Given that the molecular structure of the RAIDD DD:PIDD DD complex is available, in this study, we analyzed the molecular mechanisms underlying TLIS caused by the RAIDD TLIS variants by performing mutagenesis and biochemical assays.

## Introduction

The balance between cell proliferation and cell death is critical for normal development and homeostasis in multicellular organisms [1–4], and disruption of this balance leads to serious human diseases, such as cancers and neurodegenerative diseases [1, 5–8]. Apoptosis, a type of programmed cell death, is mediated by the sequential activation of caspases, a family of cysteine proteases that cleave specifically after aspartic acid residues [9, 10]. Caspases are divided into two classes according to their roles in apoptosis and their sequence of activation, namely, initiator caspases (including caspases 2, 8, 9, and 10) and effector caspases (including caspases 3 and 7) [10–13]. Initiator caspases are activated via the formation of huge molecular complexes, which can induce self-activation through proximity to the caspases. On the other hand, effector caspases are constitutive dimers and are activated upon cleavage by initiator caspases [14–16]. Caspase-8, -9, -1, and -2 are activated by the death-inducing signaling complex (DISC) [17], the apoptosome [18], the inflammasome [19, 20], and the PIDDosome [21], respectively, which are well-known molecular complexes required for the activation of initiator caspases.

Caspase-2, the most evolutionarily conserved caspase, is considered an initiator caspase based on its activation process. Initiator caspases contain N-terminal pro-domains that mediate protein interactions during the formation of caspase-activating complexes [22]. Caspase-2 contains an N-terminal pro-domain, known as caspase recruiting domain (CARD), which mediates protein-protein interactions to facilitate the formation of the PIDDosome, the caspase-2 activating complex. PIDDosome is composed of three proteins, namely, the p53-induced protein with a death domain (PIDD), RIP-associated Ich-1/Ced-3 homologous protein with a death domain (RAIDD), and caspase-2 [4, 21]. Upon genotoxic stress-induced apoptosis, caspase-2 is recruited to PIDD, a stress sensor protein that contains the death domain (DD), by RAIDD, an adapter protein that contains both the CARD and DD [23]. PIDDosome formation is mediated by a DD:DD interaction between PIDD and RAIDD and by a CARD:CARD interaction between RAIDD and caspase-2 [24–26]. Caspase-2 can be activated without formation of PIDDosome, indicating that alternative PIDD-independent mechanism of caspase-2 activation exists in mammals [27–29]

Recent studies have reported caspase-2 dependent cell death and related neurodegenerative diseases [30, 31]. Caspase-2-dependent neuronal cells death were detected after transient global cerebral ischemia, and inhibition of PIDDosome assembly was suggested to be an effective therapeutic approach against neuronal cell death [31]. The above findings suggested that blocking PIDDosome formation can be an effective strategy for the treatment of neurodegenerative diseases caused by excessive neuronal cell death under certain conditions [32]. Moreover, recent studies have suggested the role of caspase-2 in brain development [33]. Results of a whole-exome sequencing study showed that the RAIDD mutations in the DD, including G128R, F164C, R170C, and R170H mutations, cause thin lissencephaly (TLIS) by reducing caspase-2-mediated neuronal apoptosis [33]. Although the molecular mechanisms underlying the pathogenesis of TLIS by RAIDD variants were not identified, the findings showed that reduced caspase-2 activation in TLIS was not mediated by the loss of interaction between RAIDD variants and PIDD. Given that the molecular structure of the RAIDD DD:PIDD DD complex is available [34], we analyzed the molecular mechanisms underlying TLIS caused by the RAIDD TLIS variants (G128R, F164C, R170C, and R170H) by performing mutagenesis and biochemical assays.

## Material and methods

### Sequence alignment and molecular imaging

The amino acid sequences of RAIDD DD across different species were analyzed using Clustal Omega (http://www.ebi.ac.uk/Tools/msa/clustalo/). Molecular structure images were generated using the PyMOL Molecular Graphics System [35]

### Protein expression and purification

Previously generated clones for full-length RAIDD (1–199), RAIDD DD (94–199), and PIDD DD (777–883) were used for the current study [34]. Recombinant full-length RAIDD, RAIDD DD, and PIDD DD were expressed in *Escherichia coli* BL21 (DE3) RILP and purified as previously described [34]. Briefly, protein expression was induced by treatment with 0.5 mM isopropyl-β-D-thiogalactopyranoside (IPTG) overnight at 20°C. Bacteria were then collected, resuspended, and lysed by sonication in 80 ml of lysis buffer (20 mM Tris-HCl at pH 7.9, 500 mM NaCl, 10 mM imidazole, and 5 mM β-ME). Afterwards, cell debris were removed by centrifugation at 10,000 *g* for 1 h at 4°C. His-tagged targets were purified by affinity chromatography using Ni-NTA beads (Qiagen) and size exclusion chromatography using S-200 (GE healthcare) pre-equilibrated with buffer containing 20 mM Tris-HCl pH 8.0 and 150 mM NaCl.

### Mutagenesis

Site-directed mutagenesis of RAIDD DD was performed using the Quickchange kit (Stratagene) following the manufacturer’s protocols. Mutagenesis was confirmed by sequencing. Mutant proteins were prepared using the same method described above.

### Size exclusion chromatography assay for complex formation

To detect complex formation by size exclusion chromatography, purified full-length RAIDD, RAIDD DD, and each RAIDD mutant were mixed with a molar excess of PIDD DD and subsequently incubated for 30 min at room temperature. Samples were then run through a size exclusion column (Superdex 200 HR 10/30, GE healthcare) that was pre-equilibrated with a solution containing 20 mM Tris-HCl (pH 8.0) and 150 mM NaCl. Fractions were collected and subjected to SDS-PAGE. Coomassie Brilliant Blue was used for staining and detection of the co-migrated bands.

### Native PAGE shift assay for detecting complex formation

Protein interaction between various RAIDD mutants and PIDD DD was monitored by native (non-denaturing)-PAGE on a PhastSystem (GE Healthcare) with pre-made 8% to 25% acrylamide gradient gels (GE Healthcare). Separately purified proteins were pre-incubated at room temperature for 30 mins before loading on the gel. Coomassie Brilliant Blue was used for staining and detection of the shifted bands.

## Results

### Point mutations of the RAIDD-TLIS variants

**Fig 1:**
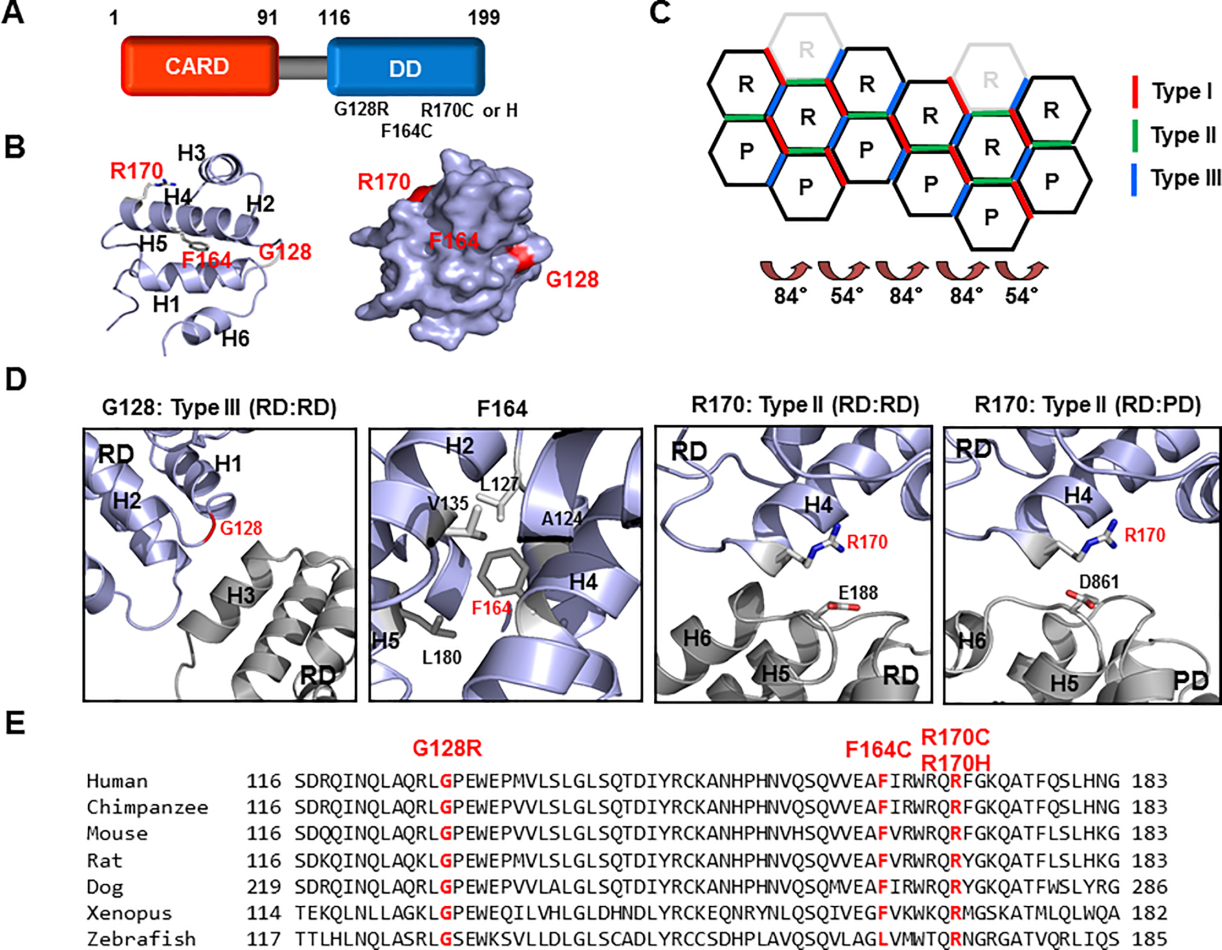
Locations of the point mutations in the corresponding RAIDD-TLIS variants. A. Domain organization of RAIDD. CARD: Caspase recruiting domain, DD: Death domain. The corresponding amino acids are shown above the protein domains. Point mutations associated with TLIS are indicated under the death domain. B. Cartoon structure of RAIDD DD. The six helix bundles are named H1 to H6. The positions of TLIS-causing point mutations on RAIDD DD are indicated in red color. Right panel shows the surface of the RAIDD DD. C. Schematic planar diagram showing the formation of the RAIDD DD:PIDD DD complex. The positions of the three different types of interfaces are shown. The angles of two different types of screw rotations are shown. R and P indicate RAIDD DD and PIDD DD, respectively. D. Protein-protein interface (PPI) and the location of each mutation on RAIDD DD in the RAIDD DD:PIDD DD complex. The residues responsible for TLIS pathogenesis are shown in red. Residues that are critical for the interactions are labelled. RD and PD indicate RAIDD and PIDD, respectively. E. Cross-species alignment of the amino acid sequences of RAIDD DD. The positions of RAIDD-TLIS variants are shown in red.

The adaptor protein for assembly of PIDDosome (RAIDD) is composed of two distinct protein interaction domains, namely, the CARD at the N-terminus and the DD at the C-terminus (Fig 1A). Both CARD and DD belong to the death domain superfamily (DDS), one of the largest protein interaction modules that also includes the death effector domain (DED) and the Pyrin domain (PYD) [4, 36]. The DDS comprises more than 100 protein members that are primarily involved in both cell death and inflammation. The DDS is characterized by a common structural fold with six anti-parallel α-helix bundles. Four TLIS-causing RAIDD variants (RAIDD-TLIS variants), namely, G128R, F164C, R170C, and R170H, have been recently identified via whole-exome sequencing [33] and were found to be located at the C-terminal DD (Fig 1A). To elucidate the molecular mechanisms underlying disease pathogenesis by the RAIDD variants, we mapped the location of each variant in the RAIDD DD. Results revealed that G128 is located in the loop connecting H1 and H2, F164 is located on H4, and R170 is located on H4 (Fig 1B). Surface mapping indicated that G128 and R170 are localized on the surface, whereas F164 is not exposed on the surface (Fig 1B). Our previous structural studies have shown that the PIDDosome core comprises five PIDD DD and seven RAIDD DD molecules that cooperatively assemble into a large oligomeric structure [34]. DDs form three layers in the complex, namely, the bottom layer comprising five PIDD DDs, the middle layer comprising five RAIDD DDs, and top layer comprising two additional RAIDD DDs, which are likely to be not necessary for complex formation [37]. This DD complex is formed via three types of interactions that are classified based on the protein regions involved in these interactions. There are eight different kinds of interfaces that are dependent on whether the interactions are between RAIDD DD and PIDD DD (R:P), between two RAIDD DDs (R:R), and between two PIDD DDs (P:P) (Fig 1C). Analysis of the positions of the RAIDD-TLIS variants in the complex showed that G128 is located in the type III interface, which is formed between H3 of the first DD and the H1-H2 and the H3-H4 connecting loops of the second DD (Fig 1D). F164 is located in the helix bundle and forms a hydrophobic cluster with neighboring hydrophobic residues (L127, V135, and L180), which could be critical for maintaining the stability of six-helix bundle fold of the DD (Fig 1D). A previous study showed that CARD, another member of the DDS, is critical for interactions in the hydrophobic cluster, which in turn maintains the stability of the fold [38]. R170 is located in the type I interface, which is formed via interactions among the H4 of the first DD and H5-H6 loop and the H6 helix of the second DD (Fig 1D). Results of inter-species alignment showed that all these residues related to the RAIDD-TLIS variants were highly conserved from humans to fish (Fig 1E), indicating that the G128, F164, and R170 residues are important for the proper function of RAIDD.

### The RAIDD DD-TLIS variants, RD-DD R170C and RD-DD R170H, failed to interact with PIDD DD *in vitro*

**Fig 2:**
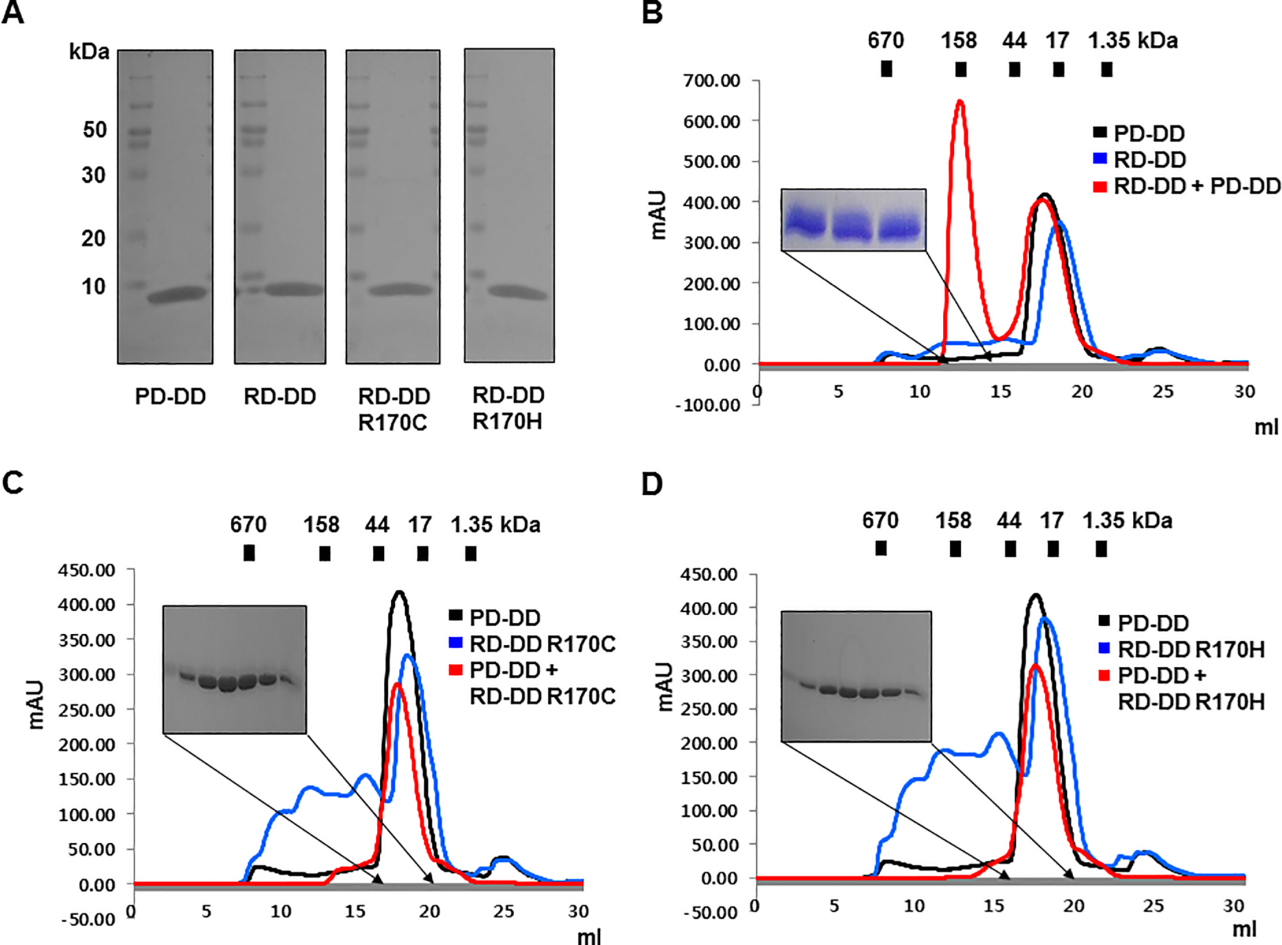
RAIDD DD-TLIS variants, R170C and R170H, fail to interact with PIDD DD *in vitro*. A. Purification of RAIDD DD, PIDD DD, and two RAIDD DD-TLIS variants. Purified protein samples prepared by two chromatography steps (affinity and size exclusion) were subjected to SDS-PAGE. Protein bands on a 15% SDS-PAGE gel were detected by Coomassie blue staining. The migration of size markers is indicated on the left side. PD-DD and RD-DD indicate PIDD DD and RAIDD DD, respectively. B. The size exclusion chromatography profile showed that wild-type RAIDD DD formed a stable complex with PIDD DD. Red peak fractions eluted at around 12–13 ml were loaded onto SDS-PAGE gels and stained with Coomassie blue. C and D. Size exclusion chromatography profiles of the mixture of PIDD DD with either RD-DD R170C variant (C) or RD-DD R170H variant (D). Red peak fractions from the mixtures of PIDD DD and two variants of RAIDD DD were loaded onto SDS-PAGE gels and visualized by Coomassie staining.

Point mutations on RAIDD that cause TLIS are specifically found on the death domain (DD), a protein-protein interaction module. Moreover, structural analysis of the RAIDD DD and PIDD DD complex showed that the point mutations G128 and R170 are located on the protein-protein interface (PPI) [34]. Thus, although cell experiments indicated that RAIDD-TLIS variants did not lose the ability to interact with PIDD, we suspected that RAIDD-TLIS variants impaired the interaction with PIDD and lost their adaptor function, which is crucial for PIDDosome formation. As a first step to elucidate the molecular basis underlying TLIS pathogenesis associated with RAIDD mutations, we attempted to express and purify RAIDD-TLIS variants and analyze their interactions with PIDD DD *in vitro*. RAIDD DD instead of the full-length RAIDD was mutated to produce the RAIDD-TLIS variants. Afterwards, the RAIDD DD of the RAIDD-TLIS variants (RD-DD G128R, RD-DD F164C, RD-DD R170C and RD-DD R170H) were expressed and purified. During the purification steps, the two variants, RD-DD G128R and RD-DD F164C, were not properly expressed and purified, whereas the expression and purification of the two other variants, RD-DD R170C and RD-DD R170H, were similar to those of the wild-type protein (Fig 2A and, S1 and S2 Figs). The amounts and purities of the final protein samples of RD-DD R170C and RD-DD R170H after two rounds of affinity and size exclusion steps were almost the same as those of the wild-type RAIDD DD and PIDD DD (Fig 2A). The unidentified impurities co-expressed with RD-DD R170C and RD-DD R170H variants were removed by size exclusion chromatography (S1A and S1B Fig). The RD-DD F164C variant was lowly expressed and purified. However, most of the proteins were detected on the nickel beads (S2A Fig) and precipitated immediately after size exclusion purification (S2B Fig). Consistent with the expected results, the above findings indicated that the point mutation in the RD-DD F164C variant led to loss of stability. The RD-DD G128R variant was not expressed at all (S2C Fig). Given the limited availability of pure protein samples for *in vitro* interaction assays, only two variants, namely, RD-DD R170C and RD-DD R170H, were analyzed for interactions with PIDD DD. Five or seven RAIDD DDs are known to interact with five PIDD DDs in the PIDDosome core [34]. Results of size exclusion chromatography indicated that the mixture of two DDs, namely, the wild-type RAIDD DD and PIDD DD, produced an elution peak at around 12–13 ml (Fig 2B). The two co-migrated bands were additionally detected by SDS-PAGE of the peak fraction, indicating that wild-type RAIDD DD forms a stable complex with PIDD DD, with a complex size of around 150 kDa (Fig 2B). The mixture of PIDD DD with RD-DD R170C or RD-DD R170H did not produce distinct complex elution peaks after size exclusion chromatography (Fig 2C and 2D), which clearly indicated that two point mutations that cause TLIS, namely, R170C and R170H, completely impaired complex formation with PIDD.

### The full-length RAIDD-TLIS variants, R170C and R170H, failed to interact with PIDD DD, while another variant, F164C, lost its stability

**Fig 3:**
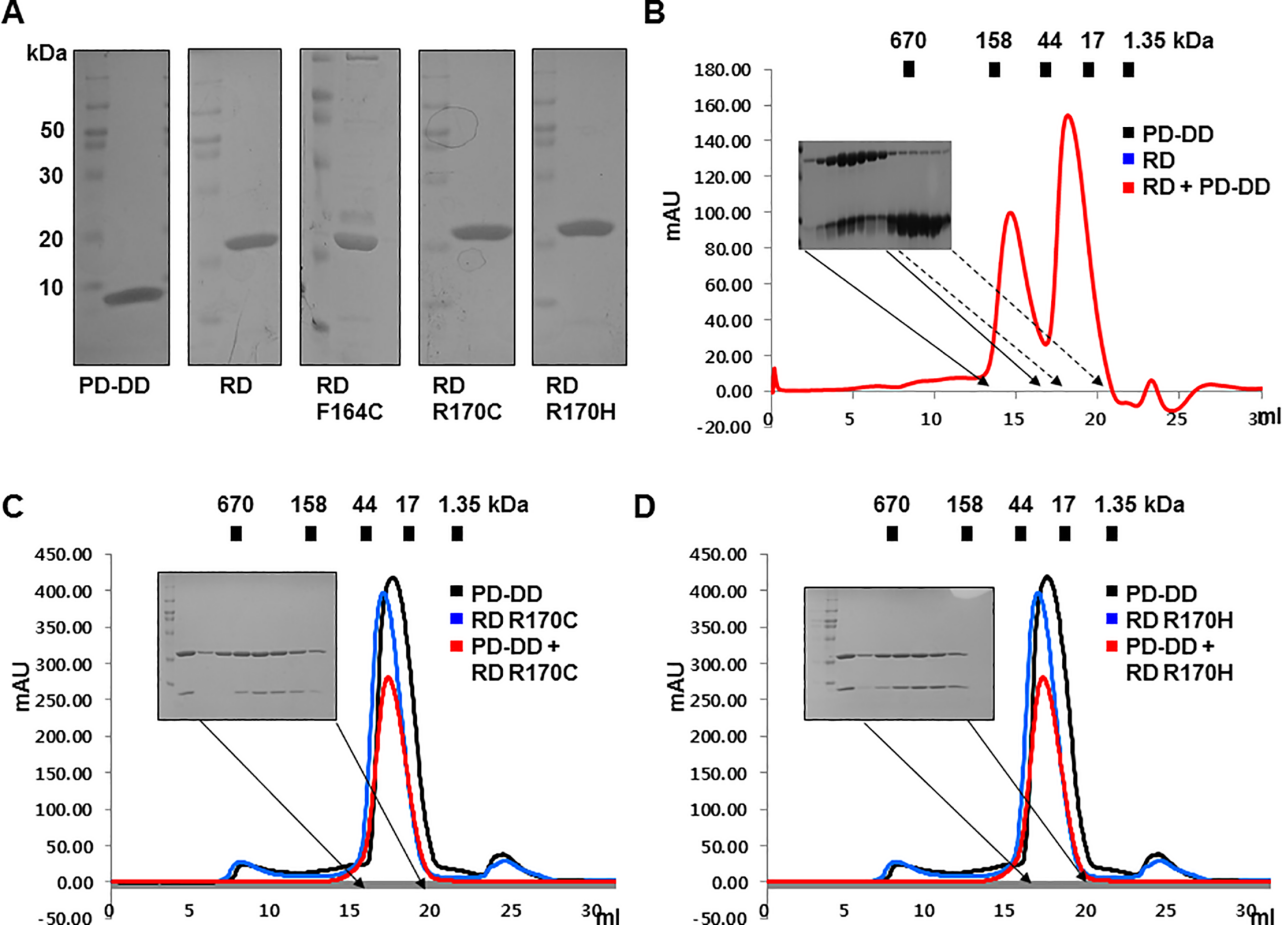
Full-length RAIDD-TLIS variants, R170C and R170H, failed to interact with PIDD DD, while another variant, F164C, lost its stability. A. Purification of full-length RAIDD, PIDD DD, and three full-length RAIDD-TLIS variants. Finally, purified protein samples were subjected to SDS-PAGE, and protein bands were detected by Coomassie blue staining. The migration of size markers is shown on the left side. PD-DD and RD indicate PIDD DD and full-length RAIDD, respectively. B. Results of size exclusion chromatography showed that wild-type RAIDD formed a stable complex with PIDD DD. Red peak fractions produced by RAIDD:PIDD DD complex that eluted at around 12–13 ml were subjected to SDS-PAGE and Coomassie blue staining. C and D. Results of size exclusion chromatography of the mixture of PIDD DD and the RD R170C variant (C) or RD R170H variant (D). Red peak fractions from the mixtures of PIDD DD and two variants of RAIDD were subjected to SDS-PAGE and visualized by Coomassie staining.

Full-length RAIDD harboring CARD and DD, which are responsible for homotypic interactions, interacts with PIDD DD [32]. Therefore, we evaluated the interactions between RAIDD-TLIS variants and full-length RAIDD and PIDD DD. To investigate these interactions *in vitro*, we mutated the full-length RAIDD construct to generate RAIDD-TLIS variants (RD G128R, RD F164C, RD R170C, and RD R170H). The mutant proteins were purified and subsequently analyzed by size exclusion chromatography. The full-length RAIDD-TLIS variants, namely, RD F164C, RD R170C and RD-DD R170H, showed similar expression and purification as that of the wild-type protein (Fig 3A). Although the RD F164C variant was strongly expressed and was obtained at high purity, most of the proteins precipitated immediately after size exclusion purification (S3A and S3B Fig), indicating that the RD F164C variant lost its stability by point mutation, similar to the results obtained for RD-DD F164C. The RD G128R variant was not expressed at all, similar to the RD-DD G128R variant (S3C Fig). Because of availability, RD R170C and RD R170H were analyzed for interaction with PIDD DD. The wild-type full-length RAIDD formed a stable complex with PIDD DD, and the peak was observed at around 12–13 ml (Fig 2B). However, the mixture of RAIDD-TLIS variants and PIDD DD failed to produce any distinct complex peak after size exclusion chromatography (Fig 3C and 3D), indicating that impaired complex formation of RAIDD-TLIS variants with PIDD was also observed in full-length RAIDD.

### Native PAGE demonstrated that RAIDD-TLIS variants cannot interact with PIDD

**Fig 4:**
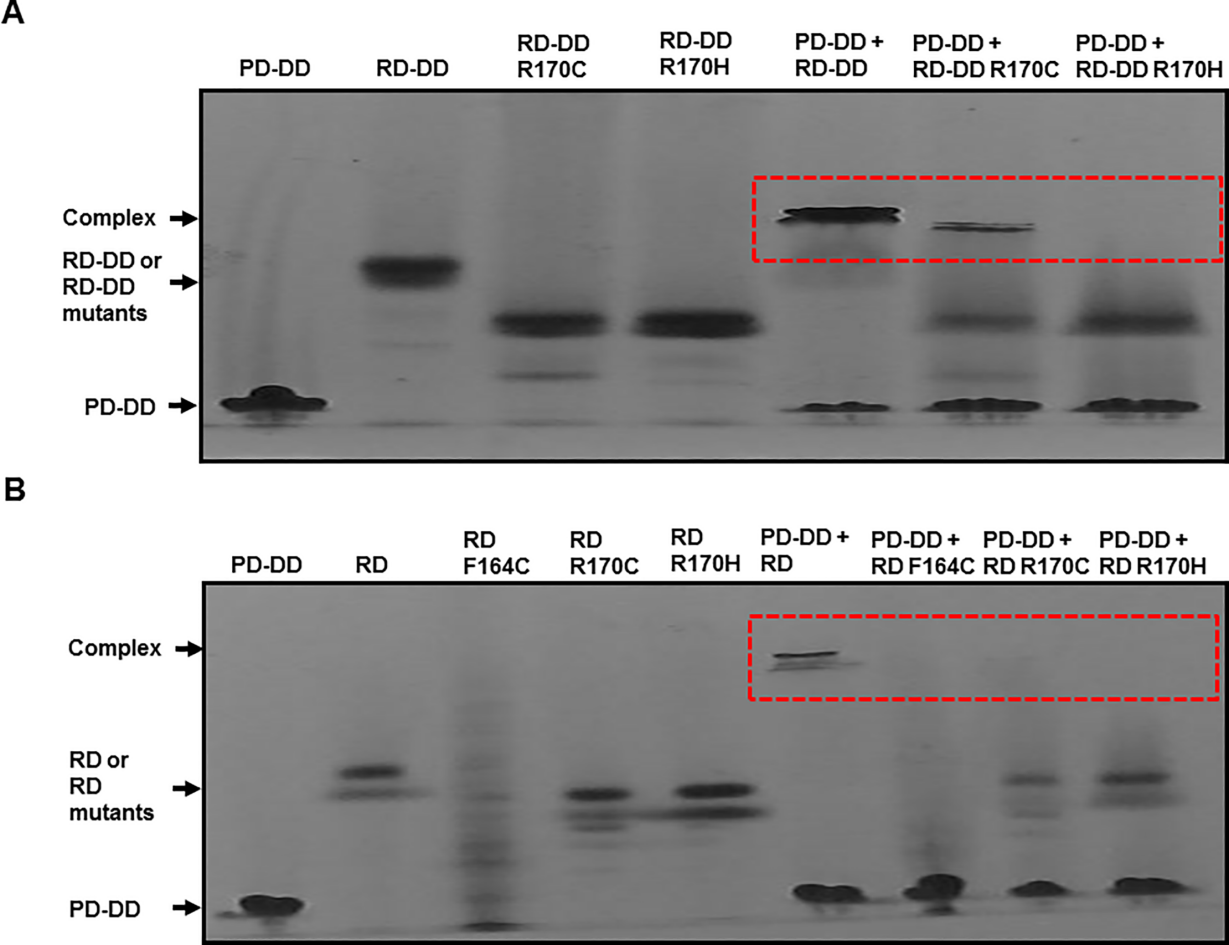
Native PAGE confirmed impaired interactions of RAIDD-TLIS variants with PIDD. A and B. Complex formation between PIDD DD and RAIDD DD-TLIS variants (A) or between PIDD DD and full-length RAIDD-TLIS variants (B) was analyzed by native PAGE. Red dotted boxes indicated newly produced complex bands.

The loss of function of RAIDD-TLIS variants on PIDD interaction was confirmed by native PAGE analysis. Bands corresponding to the two RAIDD DD variants, namely, RD-DD R170C and RD-DD R170H, were observed below the band corresponding to the wild-type RAIDD DD (Fig 4A). The mixture of RAIDD DD and PIDD DD produced a clear complex band, whereas the mixture of PIDD DD and RD-DD R170H failed to produce a complex band (Fig 4A). The amount of the complex produced by the mixture of PIDD DD and RD-DD R170C was low (Fig 4A). The above results indicated that the R170 mutation in RAIDD markedly disrupted the interaction with PIDD *in vitro*. Similar results were obtained when full-length RAIDD-TLIS variants were analyzed by native PAGE. The RD F164C, RD 170C, and RD R170H variants showed complete loss of interaction with the PIDD DD (Fig 4B). Native PAGE of the RD F164C variant produced multiple bands, which could be attributed to incorrect folding caused by the mutation (Fig 4B).

### Synthetic peptides including RAIDD-TLIS variants inhibit the interaction between RAIDD and PIDD

**Fig 5:**
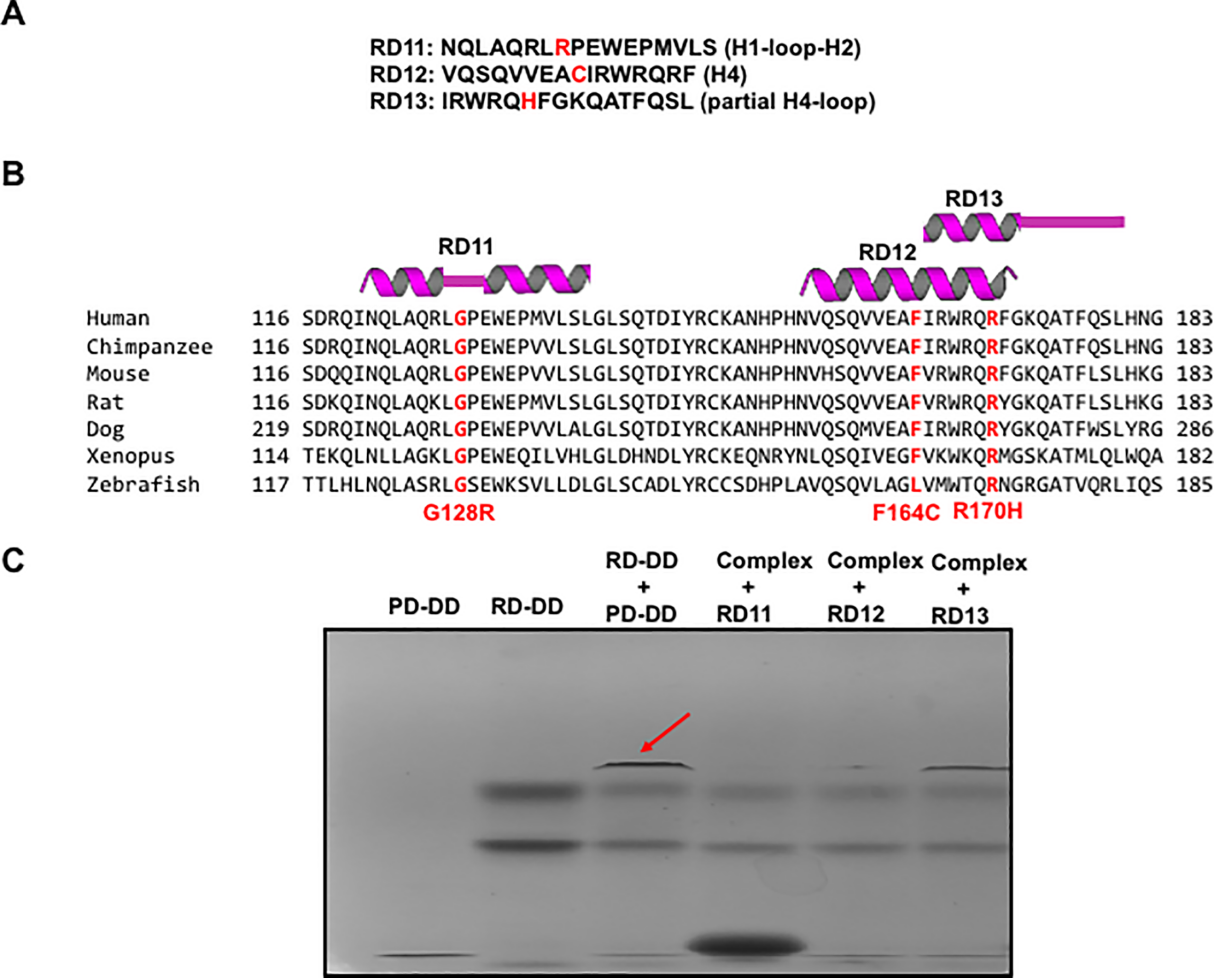
Synthetic peptides including RAIDD-TLIS variants inhibit the interaction between RAIDD and PIDD. A. Synthesized three peptides derived from RAIDD-TLIS variants. B. The location and tentative secondary structure of synthetic petides. C. Native PAGE for analyzing the interaction between RAIDD and PIDD in the presence of three peptides. Red-arrow indicates the complex band newly produced by mixing RD-DD with PD-DD. Disappearing complex band in the presence of RD11 and RD12 indicates that those peptides can block the interaction between RAIDD and PIDD.

Regional important of RAIDD binding to PIDD has been tested with synthetic peptides. Several helix peptides derived from RAIDD was blocked the interaction between RAIDD and PIDD [32, 39]. To confirm our finding that RAIDD-TLIS variants lost its ability to PIDD interaction and TLIS variant-containing region is important for protein interaction, we performed binding inhibition study with synthetic peptides including RAIDD-TLIS variants. For this experiment, we synthesized three peptides, RD11~ RD13 based on the structure and sequence alignment (Fig 4A and 4B). As shown at Fig 5C, synthetic peptides, RD11 (containing G128R variant) and RD12 (containing F164C), block the interaction of RD-DD and PD-DD. Although this result is limited to in vitro environment, this experimental result supports our conclusion that RAIDD-TLIS variants failed to bind to PIDD followed by reducing caspase-2 activity in the pathogenesis of TLIS.

## Discussion

PIDDosome formation followed by caspase-2 activation by proximity-induced self-cleavage is a critical step for programmed cell death in certain cell types, including neuronal cells. Higher oligomerization of macromolecules is considered as a critical event for various cellular signaling events [40–42]. Genotoxic stress is the most well-known trigger for caspase-2 activation. Considering the involvement of caspase-2 in neuronal cell death, blocking PIDDosome formation was suggested as an effective therapeutic intervention against neurodegenerative diseases caused by excessive neuronal cell death under certain conditions [32, 33]. A recent study that performed whole-genome sequencing of TLIS patients suggested the role of caspase-2 in the brain [33]. RAIDD mutations in the DD, namely, G128R, F164C, R170C, and R170H, were found to cause TLIS by reducing caspase-2-mediated neuronal apoptosis [33]. Given that all the TLIS-causing mutations are located in the RAIDD DD (protein interaction modules), the loss of the binding activity of RAIDD caused by mutations are likely to mediate disease pathogenesis. Previously, we solved the RAIDD DD: PIDD DD complex structure, identified all the interaction interfaces involved in complex formation [34]. In this study, we attempted to identify the effects of RAIDD DD mutations on caspase-2 activity and TLIS. Results of a mapping study showed that among the three TLIS-related residues (G128 located in the loop connecting H1 and H2, F164 located in H4, and R170 located in H4), G128 and R170 are localized on PPI (protein-protein interface), whereas F164 is located inside the six-helix bundle fold of RAIDD DD. The above results suggested that the G128R variant (located in the type III interface, which is formed between H3 of the first DD and the H1-H2 and the H3-H4 connecting loops of the second DD) and the R170C or H variants (located in the type I interface, which is formed by between H4 of the first DD and H5-H6 loop and H6 helix of the second DD), disrupted the interactions with PIDD. In turn, impaired interactions with PIDD inhibited PIDDosome formation, which is required for caspase-2 activation. On the other hand, the F164C variant potentially leads to incorrect folding of RAIDD DD because F164 is located in the helix bundle and is responsible for the formation of hydrophobic clusters with neighboring hydrophobic resides. Results of mutagenesis, size exclusion chromatography, and native PAGE experiments demonstrated that two R170 TLIS variants, R170C and R170H, lost their ability to bind PIDD DD. The F164C variant was extremely unstable, while the G128R variant was not expressed. Taken together, our current *in vitro* findings supported the notion that two RAIDD-TLIS variants, namely, R170C and R170H, cause defective interactions with PIDD. The F164C variant was found to be caused to the loss of stability of RAIDD, although cell experiments indicated that RAIDD-TLIS variants retained the ability to interact with PIDD [33]. If RAIDD-TLIS variants still bind to PIDD, there might be alternative mechanism required for caspase-2 activation in the pathogenesis of TLIS. Recent studies showed that LUBAC is essential for embryogenesis by preventing cell death and OTULIN limits cell death by deubiqutination LUBAC [43, 44]. It will be interesting to examine the tentative involvement of LUBAC and OTULIN in the RAIDD mutation-mediated TLIS pathogenesis. Although, further studies are required to explain the discrepancy between the *in vitro* and *in vivo* results, the current findings suggested that the RAIDD-TLIS variants lost its capacity to interact to PIDD or, at least, reduced the binding affinity to PIDD.

## Supporting information

S1 Fig **Purification of two RAIDD DD-TLIS variants, RD-DD R170C (A) and RD-DD R170H (B).** Size exclusion chromatography profiles are shown in the upper panel. SDS-PAGE results of fractions from size exclusion chromatography are shown in the lower panel. Black and red bars indicate impurities and target proteins, respectively.
(TIF)

S2 Fig Purification of two RAIDD DD-TLIS variants, RD-DD F164C and G128R. (A) His-tag affinity purification of RD-DD F164C. Collected fractions eluted from 250 mM imidazole are indicated by blue lines. (B) Size exclusion chromatography profiles. SDS-PAGE results of fractions from size exclusion chromatography are shown in the lower panel. Red lines indicate the eluted target proteins. (C) His-tag affinity purification of RD-DD G128R. Collected fractions eluted from 250 mM imidazole are indicated by blue lines.
(TIF)

S3 Fig Purification of two full-length RAIDD-TLIS variants, RD F164C and RD G128R. (A) His-tag affinity purification of RD F164C. Collected fractions eluted from 250 mM imidazole are indicated by blue lines. (B) Size exclusion chromatography profiles. SDS-PAGE results of fractions from size exclusion chromatography are shown in the lower panel. Red lines indicate the eluted target proteins. (C) His-tag affinity purification of RD G128R. Collected fractions eluted from 250 mM imidazole are indicated by blue lines.
(TIF)
