# Supplementary figures and images for: RAIDD mutations underlie the pathogenesis of thin lissencephaly (TLIS)
Source: PLoS One. 2018 Oct 3;13(10):e0205042. doi: 10.1371/journal.pone.0205042 (PMC6169973; doi:10.1371/journal.pone.0205042)

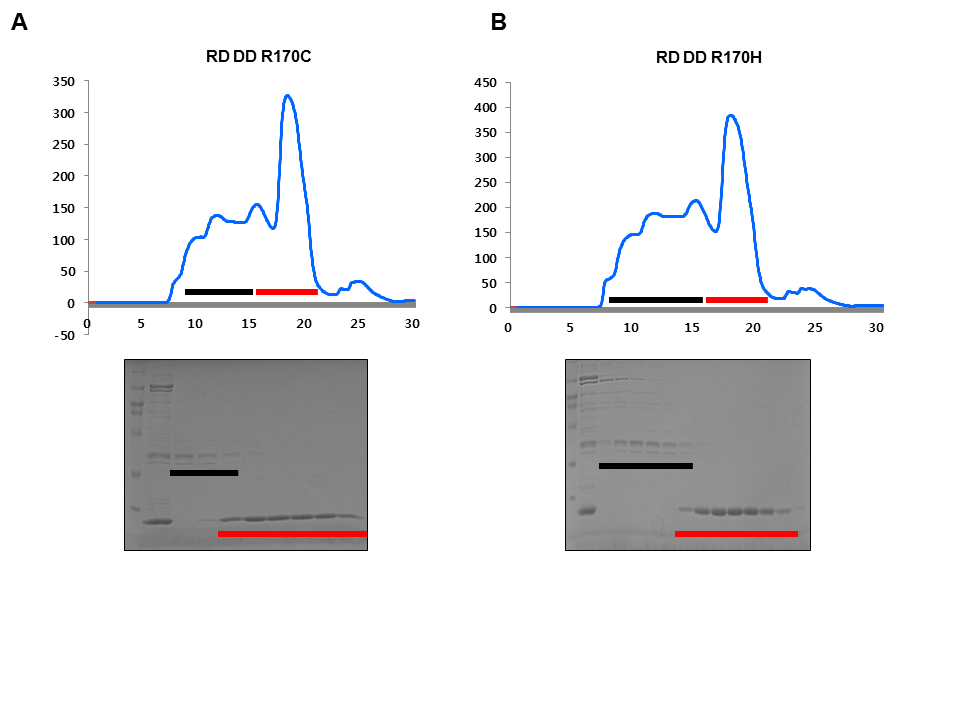

Supplement: S1 Fig — Purification of two RAIDD DD-TLIS variants, RD-DD R170C (A) and RD-DD R170H (B). Size exclusion chromatography profiles are shown in the upper panel. SDS-PAGE results of fractions from size exclusion chromatography are shown in the lower panel. Black and red bars indicate impurities and target proteins, respectively. (TIF) [file pone.0205042.s001.TIF]

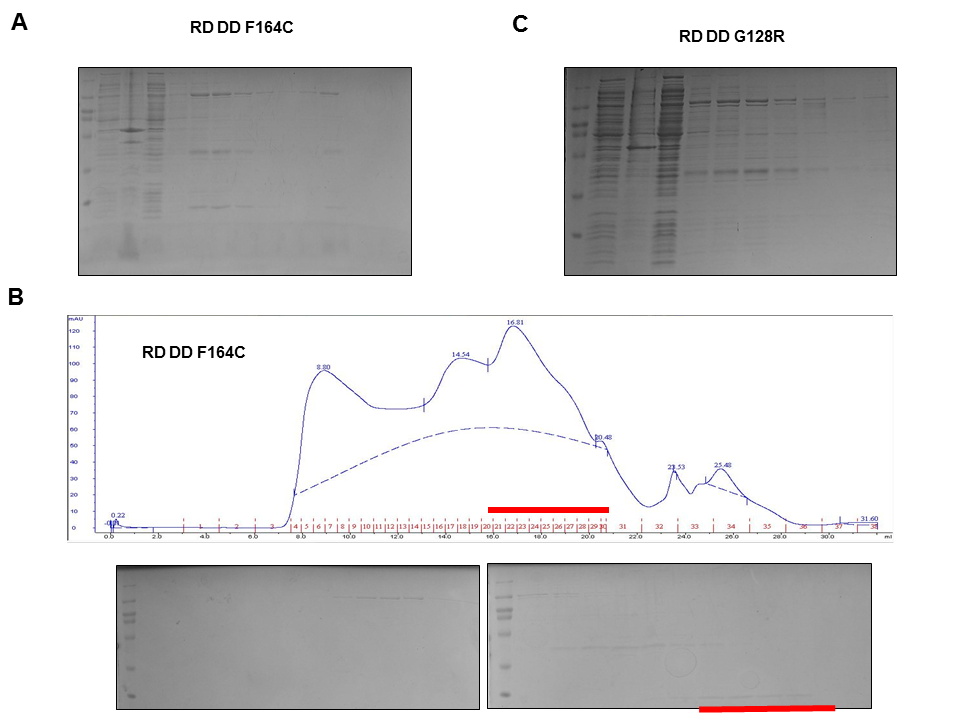

Supplement: S2 Fig — (A) His-tag affinity purification of RD-DD F164C. Collected fractions eluted from 250 mM imidazole are indicated by blue lines. (B) Size exclusion chromatography profiles. SDS-PAGE results of fractions from size exclusion chromatography are shown in the lower panel. Red lines indicate the eluted target proteins. (C) His-tag affinity purification of RD-DD G128R. Collected fractions eluted from 250 mM imidazole are indicated by blue lines. (TIF) [file pone.0205042.s002.TIF]

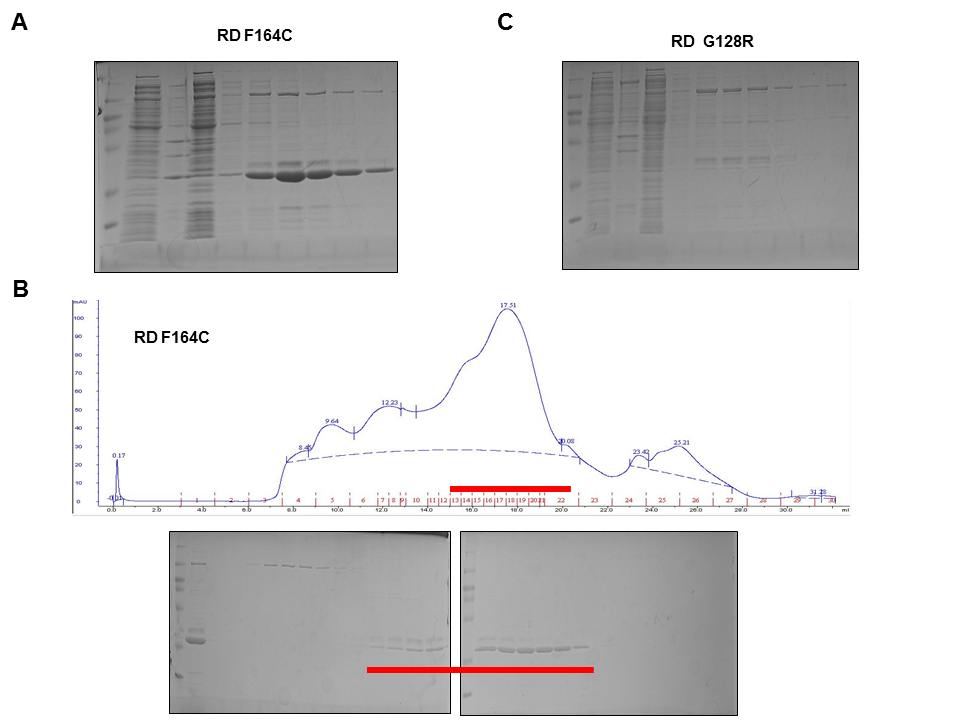

Supplement: S3 Fig — (A) His-tag affinity purification of RD F164C. Collected fractions eluted from 250 mM imidazole are indicated by blue lines. (B) Size exclusion chromatography profiles. SDS-PAGE results of fractions from size exclusion chromatography are shown in the lower panel. Red lines indicate the eluted target proteins. (C) His-tag affinity purification of RD G128R. Collected fractions eluted from 250 mM imidazole are indicated by blue lines. (TIF) [file pone.0205042.s003.TIF]
